# Safety and Efficacy of Natural and Conventional Psychiatric Treatments: A Comparative Review of Mushroom-Derived Compounds and Medicinal Plants in the Management of Mental Disorders

**DOI:** 10.3390/molecules31173130

**Published:** 2026-09-07

**Authors:** Katarzyna Gawłowska, Katarzyna Kaja Nowakowska, Julia Wiktoria Makówka, Wojciech Bajurny, Magdalena Patrycja Góral, Agata Bocheńska, Szymon Dariusz Kopecki, Weronika Marta Grodzińska, Agnieszka Chłopaś-Konowałek

**Affiliations:** 1 Scientific Society for Neurotoxicology, Department of Forensic Medicine, Wroclaw Medical University, J. Mikulicza-Radeckiego 4J, 50-345 Wroclaw, Poland; katarzyna.gawlowska@student.umw.edu.pl (K.G.); julia.makowka@student.umw.edu.pl (J.W.M.); wojciech.bajurny@student.umw.edu.pl (W.B.); magdalena.goral@student.umw.edu.pl (M.P.G.); agata.bochenska@student.umw.edu.pl (A.B.); szymon.kopecki@student.umw.edu.pl (S.D.K.); grodzinskaw@gmail.com (W.M.G.); 2 The Faculty of Medicine and Health Sciences, Collegium Medicum of the University of Zielona Gora, Zyty 28, 65-046 Zielona Gora, Poland; 108767@g.elearn.uz.zgora.pl; 3 Department of Forensic Medicine, Division of Molecular Techniques, Wroclaw Medical University, Sklodowskiej-Curie 52, 50-369 Wroclaw, Poland

**Keywords:** psilocybin, psilocin, tryptamines, *Hericium erinaceus*, muscimol, ibotenic acid, ergothioneine, psychedelic therapy, neuroprotection, depression, anxiety, neuroinflammation, psychiatric disorders, medicinal plants, *Hypericum perforatum*, *Valeriana officinalis*

## Abstract

Standard pharmacotherapy for mental disorders, including depression and anxiety, is supported by extensive clinical validation and international treatment guidelines. However, it is frequently associated with limitations such as adverse side effects, low patient adherence, and the risk of relapse. Conventional approaches rely primarily on antidepressants, anxiolytics, mood stabilizers, neuroleptics, and stimulants, which modulate monoaminergic, GABAergic, and dopaminergic neurotransmission. Recently, preclinical and emerging clinical studies have suggested that selected natural substances—specifically mushroom-derived compounds and well-established medicinal plants—may offer complementary therapeutic pathways. This narrative review critically evaluates the pharmacological efficacy, mechanisms of action, and safety profiles of selected natural compounds, including psilocybin-related tryptamines, *Hericium erinaceus*, ibotenic acid/muscimol from *Amanita muscaria*, ergothioneine, as well as notable botanical agents (e.g., *Hypericum perforatum*, *Valeriana officinalis*). We systematically distinguish between robust human clinical data (e.g., psilocybin-assisted therapy) and preliminary in vitro/in vivo findings. Particular attention is paid to their neurotrophic, anti-inflammatory, and neuromodulatory properties, while emphasizing substantial safety concerns, including intoxication risks associated with isoxazole derivatives, and potentially severe cytochrome P450-mediated drug–drug interactions. Ultimately, while certain natural compounds show promise as adjunctive interventions, most remain strictly investigational and cannot replace evidence-based conventional pharmacotherapy without further long-term, large-scale randomized controlled trials.

## 1. Introduction

Mental disorders constitute a heterogeneous group of conditions characterized by disturbances in cognition, emotional regulation, or behavior. According to the *Diagnostic and Statistical Manual of Mental Disorders, Fifth Edition* (DSM 5), a mental disorder is defined as “a syndrome characterized by clinically significant disturbance in an individual’s cognition, emotion regulation, or behavior, reflecting a dysfunction in the psychological, biological, or developmental processes underlying mental functioning” [1]. Similarly, the International Classification of Diseases (ICD 11) describes mental, behavioral, and neurodevelopmental disorders as conditions associated with significant impairment in personal, social, educational, or occupational functioning [2].

A wide range of psychiatric disorders has been identified, including anxiety disorders, mood disorders (major depressive disorder, bipolar disorder, cyclothymia), psychotic disorders such as schizophrenia, eating disorders (anorexia nervosa, bulimia nervosa), obsessive–compulsive disorder (OCD), post-traumatic stress disorder (PTSD), impulse control disorders, substance use disorders, and personality disorders [3].

Epidemiological data from the World Health Organization indicate that mental disorders are increasing globally. Approximately 20% of children and adolescents experience a mental health condition, and suicide is the second leading cause of death among individuals aged 15–29 years [4]. Among adults, depressive and anxiety disorders remain the most prevalent, affecting more than 300 million and 280 million people worldwide, respectively [5]. In Europe, mental health disorders affect an estimated 165 million individuals annually, with anxiety disorders, mood disorders, and substance use disorders being the most common [5,6].

The treatment of mental disorders—particularly depression and anxiety—relies primarily on pharmacotherapy involving five major classes of psychotropic medications: antidepressants, anxiolytics/hypnotics, mood stabilizers, antipsychotics, and stimulants/nootropics. Commonly prescribed agents include selective serotonin reuptake inhibitors (SSRIs; e.g., escitalopram, sertraline), serotonin–noradrenaline reuptake inhibitors (SNRIs), tricyclic antidepressants (TCAs), benzodiazepines, lithium, atypical antipsychotics (e.g., olanzapine, aripiprazole), and stimulants such as methylphenidate [7,8]. Psychotherapy—particularly cognitive behavioral therapy (CBT)—is frequently used as a complementary treatment.

Despite their well-established efficacy, extensive clinical validation, and fundamental inclusion in international treatment guidelines, standard pharmacotherapies are associated with certain limitations [9,10]. These conventional medications remain the absolute cornerstone of modern psychiatry, offering undeniable clinical benefits in achieving symptom remission, preventing relapse, and significantly reducing severe psychiatric outcomes, including suicidality. However, studies demonstrate a high prevalence of adverse side effects—such as gastrointestinal disturbances, metabolic complications, sexual dysfunction, emotional blunting, and sedation—which can substantially compromise patient’s quality of life. These adverse profiles significantly contribute to poor medication adherence and premature treatment discontinuation [9,10]. For example, nearly 40% of patients discontinue antidepressant treatment within the first two months, and up to 87% within one year, often due to intolerable side effects or insufficient therapeutic response [9]. These clinical realities highlight the supplementary need for alternative or adjunctive therapeutic strategies that possess novel mechanisms of action, faster onset of clinical effects, and improved tolerability profiles, without negating the established value of conventional medicine.

To illustrate the key differences between conventional pharmacotherapy and emerging mushroom-derived compounds, Table 1 summarizes their mechanisms, clinical characteristics, and potential advantages.

Growing interest in complementary and integrative approaches has drawn attention to biologically active compounds derived from mushrooms. Traditional, complementary, and integrative medicine (TCIM) approaches—including medicinal plants and fungi—are increasingly recognized for their accessibility, lower cost, and potentially more favorable safety profiles. The World Health Organization has emphasized the importance of developing high quality natural products for mental health applications [11].

Among mushroom-derived compounds, several groups have shown promising neuropsychiatric potential. Tryptamines such as psilocybin, psilocin, baeocystin, aeruginascin, and norbaeocystin act as potent 5 HT2A receptor agonists and have demonstrated rapid and sustained antidepressant effects in clinical studies [12,13]. *Hericium erinaceus* contains hericenones and erinacines that stimulate nerve growth factor (NGF) synthesis and exhibit neuroprotective, pro-neurogenic, and antidepressant properties in preclinical and early clinical studies [14,15]. Ibotenic acid and muscimol from *Amanita muscaria* modulate NMDA and GABAA receptors and have been investigated for their anxiolytic and neuroactive properties, although concerns regarding toxicity remain [16]. Ergothioneine, a naturally occurring thiol-based antioxidant, has been associated with reduced neuroinflammation and a lower severity of depressive symptoms, suggesting a potential role in mood regulation [17].

Notably, a randomized controlled trial by Carhart Harris et al. [12] involving 59 patients demonstrated that psilocybin-assisted therapy produced antidepressant effects comparable to escitalopram, with a more favorable tolerability profile. Additional studies indicate that even a single session of psilocybin-assisted psychotherapy may induce rapid and sustained improvements in mood and emotional well-being, as demonstrated by Davis et al. [18], who reported that two high-dose psilocybin sessions administered to patients with major depressive disorder produced large and clinically significant reductions in depressive symptoms within one week, with approximately 71% of participants showing a ≥50% decrease in depression severity and 54% meeting criteria for remission at the four-week follow-up. Although research on β carbolines, ibotenates, and ergothioneine remains at an earlier stage, preclinical findings suggest potential applications in depression, anxiety, and addiction [19].

The aim of this paper is to critically compare the clinical efficacy and safety of mushroom-derived compounds and selected medicinal plants with standard pharmacological treatments for selected mental disorders, and to provide a comprehensive overview of their pharmacological properties, mechanisms of action, and potential therapeutic applications.

## 2. Review Methodology

This narrative review was structured to synthesize and critically appraise current evidence regarding mushroom-derived bioactive compounds and selected medicinal plants with potential relevance to psychiatric disorders. To ensure scientific rigor and minimize selection bias, a systematic search strategy was employed across three major biomedical databases: PubMed/MEDLINE, Scopus, and ScienceDirect. The literature search encompassed articles published between January 2000 and December 2025.

The search strategy utilized Boolean operators combining primary terms: (“psilocybin” OR “psilocin” OR “tryptamines” OR “*Hericium erinaceus*” OR “muscimol” OR “ibotenic acid” OR “ergothioneine” OR “*Hypericum perforatum*” OR “medicinal plants”) AND (“psychiatric disorders” OR “depression” OR “anxiety” OR “neuroprotection” OR “neuroinflammation” OR “clinical trial” OR “toxicity”).

Inclusion and Exclusion Criteria:

Studies were selected based on predefined eligibility criteria. Inclusion criteria comprised: (1) human randomized controlled trials (RCTs) and open-label clinical studies evaluating psychiatric outcomes; (2) human observational and epidemiological studies, including pharmacovigilance and poison control center data; (3) in vivo animal models assessing relevant behavioral and neurobiological parameters; and (4) in vitro and ex vivo mechanistic studies characterizing receptor binding profiles (e.g., 5-HT2A, NMDA, GABA-A) and neurotrophic modulation. Exclusion criteria included non-peer-reviewed literature, opinion pieces, studies lacking clear methodological frameworks, and research focusing exclusively on non-psychiatric somatic conditions.

A critical appraisal approach was actively applied to explicitly distinguish between clinically validated efficacy and preliminary preclinical hypotheses. Data regarding study design, sample size, intervention dosing, primary outcomes, and adverse events were systematically extracted and categorized. A dedicated focus was placed on identifying toxicological risks, particularly concerning *Amanita muscaria* isoxazole alkaloids and CYP450-mediated botanical interactions.

## 3. Current Limitations of Standard Pharmacological Treatments for Psychiatric Disorders

Population-based analyses have demonstrated a substantial increase in the use of prescription psychotropic medications over time, including antidepressants and antipsychotics, indicating a growing reliance on pharmacological management of mental disorders [20]. Worldwide, psychotropic medicine sales increased from 28·54 defined daily doses (DDD) per 1000 inhabitants per day in 2008 to 34·77 DDD per 1000 inhabitants per day in 2019—a relative average increase of around 4%, especially in high-income countries [21]. During the COVID-19 pandemic, the psychotropic medication consumption continued to grow [22]. In Europe, antidepressant consumption reaches approximately 60–100 defined daily doses (DDD) per 1000 inhabitants per day, while antipsychotic use remains in the range of 8–15 DDD/1000/day, reflecting a substantial and sustained scale of psychotropic medication use over recent years [23].

These drugs have a wide range of usage, often going beyond psychiatric conditions, with satisfactory performance. However, many of them come with a broad spectrum of both somatic and psychological adverse effects. What is more, in some cases, they are unfortunately insufficient for the management of illnesses [24,25]. In order to list and summarize the potential problems with the usage of conventional psychotropic drugs, we classified them into the following five groups: antidepressants, anxiolytics and hypnotics, normothymic drugs (mood stabilizers), antipsychotics and stimulants with nootropics [26].

The most often prescribed psychotropic drugs worldwide in adults of all ages are antidepressants, with selective serotonin reuptake inhibitors (SSRIs) at the forefront, due to their big safety and tolerability profile. Commonly used SSRI drugs are escitalopram, citalopram, sertraline or fluoxetine [27]. Other prescribed drugs are serotonin–noradrenaline reuptake inhibitors, noradrenaline and specific serotonergic antidepressants (NASSAs), tricyclic antidepressants, serotonin antagonists and reuptake inhibitors (SARIs) and monoamine oxidase inhibitors (MAOIs). However they are used significantly less frequently—they are reserved for more severe cases, or in case of lack of effect of first-line treatment [28]. Each year, the incidence of depressive disorders continues to rise [21,29], and yet it is believed that many cases are underdiagnosed. Because of that, the usage of antidepressants increases and with them the prevalence of their adverse effects, which are especially dangerous when combined with low adherence and poor attention to proper management aligned with international guidelines [30,31,32].

For the purpose of in-depth understanding of type and frequency of complications associated with the usage of conventional antidepressants, a selection of recent wide cohort studies among various populations is stated in Table 2.

Based on data presented in Table 2, specifically the significant rate of treatment discontinuation due to adverse effects [9], a high prevalence of some adverse effects (around 50–60%) [9,10,35], and the risk of occurrence of life-threatening complications—upper gastrointestinal bleeding [33], suicidality [10], increase in cardiovascular disease mortality [34]—it is apparent that current pharmacological therapies have limitations. These restrictions indicate the areas where alternative methods of treatments could be developed to overcome these disadvantages and ensure treatment safety and efficacy.

First of all, there is a long list of physical side effects, which, asides from the ones mentioned in the table, may also include gastrointestinal effects, such as diarrhea or constipation, neutropenia and other hematological complications, hyponatremia or serious serotonin syndrome [36]. In addition, patients can suffer from psychological problems, from which the most severe issue is suicidality, which may happen in the early stages of treatment with some of the drugs [37]. What is more is that a significant number of patients discontinue the treatment despite not feeling better due to various reasons, with the most prevalent being perceived ineffectiveness or not tolerable adverse effects [7,38,39].

Antipsychotic drugs, also known as the neuroleptics fall into two categories: atypical antipsychotics, such as olanzapine or aripiprazole and typical antipsychotics, like haloperidol and chlorpromazine—the first substance observed to have antipsychotic effects [40]. These medications are used to treat psychosis, schizophrenia and schizoaffective and bipolar disorders [41].

Antipsychotic medications are potent dopamine D2 receptor antagonists, making them especially effective in alleviating psychotic symptoms—these drugs represent the main pharmacological treatment strategy; however, they can induce several adverse effects [42]. In a cohort study published by Hynes et al. it was observed that the most commonly reported side-effects were daytime drowsiness (75%), dry mouth (58.2%) and weight gain (50.0%), while the most concerning adverse effects reported were erectile dysfunction (35.0%), sexual dysfunction (26.3%) and amenorrhea (26.3%) [43].

More severe adverse effects are extrapyramidal symptoms, such as pseudoparkinsonism, a reversible syndrome characterized by tremors in the hands and arms, muscle rigidity, bradykinesia, akinesia and reduced facial expressions. In addition, patients might feel an inability to remain still, known as akathisia. Spastic muscle contractions can occur as well, including symptoms such as oculogyric crisis (upward deviation of the eyes), retrocollis (backward neck arching), and laryngospasm (involuntary closure of the airway). In long-term antipsychotic treatment, tardive dyskinesia is observed—involuntary movements disorder, usually in the orofacial region. It may consist of myoclonic jerks, tics, choreiform motions, and dystonic postures. These symptoms cause discomfort and social stigma to the patients [44].

Anxiolytics are aimed at managing anxiety, emotional tension and panic disorders [45], and hypnotics are used to treat insomnia, which is closely related to the above-mentioned health problems [46].

Common substances included in these groups are benzodiazepines (such as diazepam, clonazepam or alprazolam), z-drugs (zaleplon, zolpidem or zopiclone) and barbiturates. These drugs are most frequently prescribed to the elderly patients; however, recent years show an increase in anxiety incidence and prescription of these medications [47,48]. That is a problematic issue, due to their high potential for addiction and abuse. The physical and psychological dependence and the possible development of withdrawal syndrome (e.g., irritability, insomnia, tremors and seizures, confusion, hallucinations) can even be fatal [49]. The misuse of BZD is especially concerning due to severe, life-threatening toxicity symptoms [47]. Abuse of these drugs by the elderly increases the risk for dementia and other types of cognitive impairment, as well as elevates the risk of falls and fractures—from which the hip fractures are most common and dangerous and are one of the “geriatric giants” [50,51].

Mood stabilizers comprise different pharmacological agents, including lithium, antiepileptic drugs such as carbamazepine, lamotrigine and valproate, and some antipsychotics as well. Lithium is the first-line treatment of bipolar disorder. However adverse reactions from different organs may appear—renal (polyuria, nephropathy), gastrointestinal, neurological (tremor), thyroid, metabolic (weight gain, alterations in calcium and bone metabolism), cognitive, dermatological (psoriasis), cardiovascular (sinus bradycardia), and sexual dysfunction. In a cohort study Pahwa et al. describes that among 154 patients with bipolar disorder who received long-term lithium therapy (LTLT), 41 patients (27%) developed CKD, of whom 20 (49%) patients continued lithium while 19 (46%) discontinued it. The median time to stage 3 CKD development was 21.7 years from initiation of the treatment [52].

Lamotrigine is a widely-used antiseizure medication, commonly prescribed for the treatment of epilepsy and bipolar disorder, and is generally well tolerated; nevertheless, large-scale studies about dangers of its use were carried out. Christensen et al. [53] published an Danish population-based cohort study, following cohort members aged ≥15 years for the first 2 years after they initiated lamotrigine therapy. There were 91.949 (36.618 males [39.8%]) new users of lamotrigine (median age = 45.7 years, interquartile range = 32.0–60.2 years). Among users without pre-existing cardiac disease (*n* = 86.769), 194 (0.23%) developed a cardiac conduction disorder. Comparison of the risk in current and past lamotrigine treatment periods yielded an adjusted HR of new onset cardiac conduction disorder of 1.03 (95% CI = 0.76–1.40). Among users with pre-existing cardiac disease (*n* = 5180), 1150 (22.2%) died. Comparison of the risk in current and past lamotrigine treatment periods yielded an adjusted HR for all-cause mortality of 1.05 (95% CI = 0.93–1.19) [54].

Valproate is an antiseizure medication associated with an increased risk of congenital malformations when used during pregnancy. In the article by Christensen et al. [54], among the 895.507 children (males, 51.3%), 31.790 (3.6%) were diagnosed with a major congenital malformation in the first year of life. In the analyses including children born in 1997, the risk of major congenital malformations among children prenatally exposed to valproate compared with children not exposed to ASMs was increased by a fully adjusted OR (aOR) of 3.95 (95% CI = 1.65–9.47). With the addition of data from the following years, the teratogenic effect of valproate was further substantiated, as the precision of the estimate improved (1997–2014: aOR = 2.44, 95% CI = 1.80–3.30) [54].

Stimulants are a group of medications used to elevate the activity level of the central nervous system. The most important psychiatric relevant medical indication for their usage is management of attention deficit hyperactivity disorder—ADHD. Most prevalent substances include amphetamines and methylphenidate [55,56]. Although this disease is primarily associated with the pediatric population, the literature indicates that it can rarely transfer into adulthood, and is often associated with other psychiatric ailments [57]. Methylphenidate is most typically prescribed to manage ADHD; however, not all adults tolerate it adequately [58]. It is linked to potential development of sleep issues, reduced appetite and possible elevated risk of cardiovascular incidents [59,60]. Moreover, stimulant use, mainly amphetamines, may induce psychosis [61].

Considering the presented information, psychotropic medication, while highly effective, is associated with several complications. Therefore, it is worth exploring alternative treatments for various psychiatric conditions to improve drug tolerability, reduce psychiatric stigma, minimize adverse effects, and enhance individualized patient care.

This article presents several psychedelic substances that show promise in treating certain psychiatric disorders.

## 4. Medicinal Plants in Psychiatry

Conventional pharmacological treatments for psychiatric disorders including antidepressants, anxiolytics, mood stabilizers, and antipsychotics remain the cornerstone of modern psychiatry. However, as outlined in Chapter 3, these therapies are frequently associated with adverse effects, tolerance development, and high discontinuation rates, which limit their long-term effectiveness [9,10]. Many patients experience diminishing therapeutic response over time, leading to dose escalation and increased medication burden. These limitations have intensified interest in alternative therapeutic strategies that may offer improved tolerability, multimodal mechanisms of action, and broader applicability.

Traditional, complementary, and integrative medicine includes a wide range of non-conventional approaches, among which medicinal plants play a central role. Herbal preparations contain diverse phytochemicals such as flavonoids, alkaloids, terpenoids, and phenolic acids—that exert neuroactive, anti-inflammatory, antioxidant, and neuromodulatory effects relevant to psychiatric disorders [62]. Their widespread use is driven by accessibility, lower cost, cultural acceptance, and a generally favorable safety profile compared with conventional psychotropic medications [63]. International organizations, including the World Health Organization, emphasize the importance of developing high quality, evidence-based herbal products and integrating them into healthcare systems where appropriate [11].

Herbal therapies are particularly recommended as adjunctive treatments or may have potential for managing mild to moderate symptoms, including subclinical depression, generalized anxiety, sleep disturbances, and stress-related conditions [62]. Compared with standard pharmacotherapy, plant-based preparations are often perceived as safer and better tolerated, with lower risks of dependence and fewer systemic side effects. Their multimodal mechanisms targeting neurotransmission, neurotrophic pathways, oxidative stress, and inflammatory signaling align with contemporary models of psychiatric pathophysiology.

This chapter discusses the therapeutic potential of selected medicinal plant species used in the management of psychiatric symptoms, with emphasis on their phytochemical composition, mechanisms of action, and available clinical evidence. To provide an overview of the most widely-used herbal agents in mental health care, Table 3 summarizes selected medicinal plants, their primary bioactive compounds, mechanisms of action, and documented therapeutic effects.

### Safety Considerations and Pharmacokinetic Drug Interactions of Medicinal Plants

While medicinal plants are often perceived by the general public as uniformly safe, they can precipitate severe, clinically significant drug–drug interactions [70]. *Hypericum perforatum* (St. John’s wort) represents a primary paradigm of this risk. Although it possesses documented clinical efficacy for mild-to-moderate depression, its active preparations are potent activators of the pregnane-X-receptor (PXR). This activation leads to a profound induction of the Cytochrome P450 enzyme system—most notably the CYP3A4, CYP2C9, and CYP1A2 isoenzymes—as well as the P-glycoprotein (P-gp) efflux transporter [71].

This enzymatic induction significantly accelerates metabolism and reduces the systemic bioavailability of numerous critical conventional medications, including oral contraceptives, immunosuppressants (e.g., cyclosporine, tacrolimus), anticoagulants (e.g., warfarin), and antiretroviral agents (e.g., indinavir), potentially leading to catastrophic therapeutic failures. Furthermore, because St. John’s wort pharmacodynamically increases the synaptic levels of serotonin, dopamine, and norepinephrine via reuptake inhibition, its concurrent use with conventional selective serotonin reuptake inhibitors (SSRIs)—such as sertraline, escitalopram, or paroxetine—creates a severe interaction [72]. This combination can dangerously elevate central serotonin levels, precipitating serotonin syndrome—a potentially life-threatening condition characterized by altered mental status, autonomic instability (hyperthermia, tachycardia), and neuromuscular abnormalities (hyperreflexia, myoclonus).

## 5. Mushroom-Derived Compounds

### 5.1. Hercium erinaceus

*Hericium erinaceus*, also known as Lion’s Mane mushroom, is naturally found in Asia, Europe, and North America [73]. This fungus has been used for centuries in traditional practice, particularly in Chinese medicine, for its therapeutic properties [74]. Contemporary research has revealed that *H. erinaceus* exhibits a broad spectrum of biological activities, which can be attributed to its unique composition of bioactive compounds. According to the literature, in addition to macromolecules such as polysaccharides and proteins, *H. erinaceus* also contains various micromolecules, including terpenoids, cerebrosides, phenols and sterols [75]. These compounds are associated with several biological effects, including anti-cancer, antioxidant, anti-inflammatory and gastrointestinal protective properties [76].

Moreover, *H. erinaceus* has been shown to exert neuroprotective effects, suggesting potential applications in the prevention and delay of progression of Alzheimer’s disease and other neurodegenerative disorders such as Parkinson’s disease, Huntington’s disease, amyotrophic lateral sclerosis (ALS), multiple sclerosis (MS), and Creutzfeldt–Jakob disease [77,78]. Of particular importance in the context of neuroprotection are hericenones and erinacines, found in the fruiting bodies and mycelia of *Hericium erinaceus*, respectively [78,79,80,81,82]. These compounds promote the synthesis of nerve growth factor (NGF) in astrocytes, contributing to the protection of brain neurons [82,83,84,85]. Studies also indicate the antidepressant and anti-inflammatory effects of amycenon, an extract derived from the fruiting body of *Hericium erinaceus*, containing 0.5% hericenone and 6% amyloban [86]. Yao et al. confirmed these properties in their study by administering amycenon to a mouse model of depression induced by lipopolysaccharide (LPS)-induced inflammation [84].

Numerous studies conducted on animal models indicate the potential of *Hericium erinaceus* in the treatment of psychiatric disorders, particularly depression and anxiety [85,86,87,88]. Chiu et al., [85] in their study conducted on mice, observed that supplementation with *Hericium erinaceus* contributed to the normalization of behavioral disturbances induced by restraint stress. They concluded that these effects were attributable to the influence of *H. erinaceus* on three key antidepressant mechanisms: restoration of hippocampal monoamine neurotransmitter levels, inhibition of plasma pro-inflammatory cytokines, and modulation of the PI3K/Akt/GSK-3β signaling pathway, resulting in increased expression of brain-derived neurotrophic factor (BDNF) [85]. In a related study, Chong et al. [86] investigated the effects of *Hericium erinaceus* in mice subjected to chronic restraint stress, using both *H. erinaceus* and temozolomide—a neurogenesis inhibitor. Interestingly, the administration of temozolomide, by blocking neurogenesis, completely abolished the antidepressant effects of *H. erinaceus*. These findings support the conclusion that the antidepressant properties of *H. erinaceus* are mediated through stimulation of neurogenesis and reduction in neuroinflammation via enhancement of the BDNF-TrkB-CREB signaling pathway [86].

Clinical studies investigating *Hericium erinaceus* typically administer 500–3000 mg/day of standardized extract. These doses have been associated with improvements in cognitive function, mood, and anxiety symptoms in both healthy adults and individuals with mild cognitive impairment [15,73].

A particularly interesting study was conducted by Nagano et al., who demonstrated a difference in scores on the Center for Epidemiologic Studies Depression Scale (CES-D) before and after a 4-week administration of *Hericium erinaceus* or placebo in a randomized cohort of 30 women divided into two groups. It was found that, despite no significant differences in CES-D scores between the two groups at baseline, only the group receiving *Hericium erinaceus* showed a significantly lower CES-D score after 4 weeks compared to pre-trial levels (*p* = 0.033) [89].

### 5.2. “Minor” Psilocybin-Related Tryptamines: Baeocystin, Norbaeocystin, Aeruginascin

**Figure 1:**
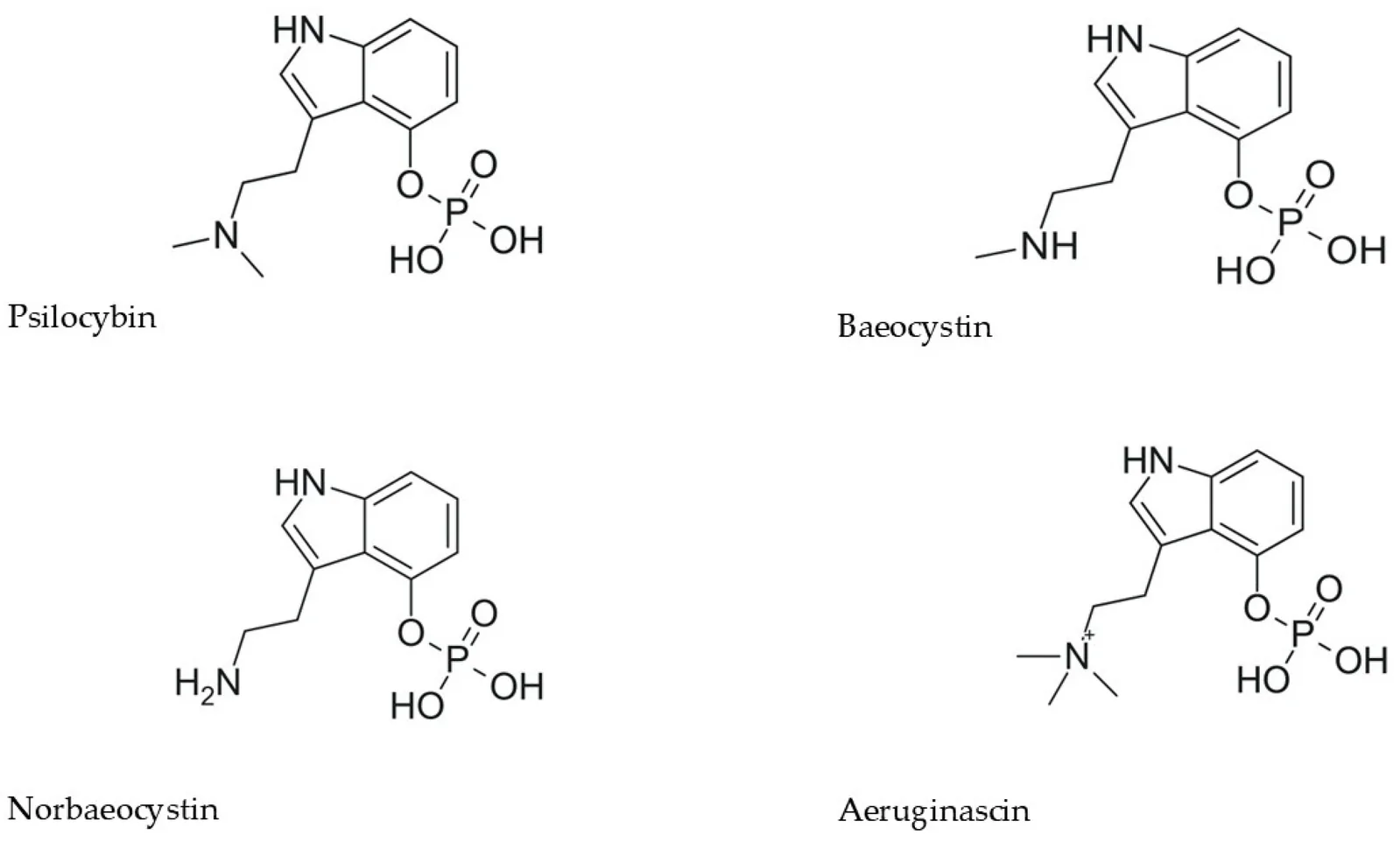
The chemical structures of the discussed tryptamines.

Psilocybin, the most famous tryptamine alkaloid found in ‘’magic’’ mushrooms, has been extensively examined for its potential therapeutic value in mental health disorders, such as depression, addiction, obsessive–compulsive disorders, post-traumatic stress, bipolar disorder and many more [90,91,92]. Subsequent studies identified and characterized several tryptamines structurally related to psilocybin, including baeocystin, norbaeocystin, and aeruginascin (Figure 1) [90]. Variable concentrations of these compounds are contained in mushroom species of the genera Gymnopilus, Conocybe, Inocybe, Mycena, Panaeolus, Pluteus, Pholiotina, and Psilocybe [91,93,94]. The chemical structure of these tryptamine alkaloids can be generalized as a molecule with the phosphate substitution at the 4-position of the indole ring and sequentially increasing degrees of N-methylation at the terminal nitrogen [95]. Because their structure resembles the psilocybin one, it may suggest that their properties, and thus the clinical utility are also similar. Unfortunately, the pharmacodynamics and pharmacokinetics of these substances have not been well studied yet and no clinical trials testing the efficacy of these tryptamines have been performed.

It is known that after ingesting “magic” mushrooms, psilocybin is dephosphorylated by alkaline phosphatase (AP) to its more active and lipophilic form, psilocin, which passively crosses the blood–brain barrier (BBB) and interacts with central target receptors. Psilocin is then metabolized by monoamine oxidase A (MAO-A) into inert metabolites [90]. The study conducted by Rakoczy et al. indicates that analogously to psilocybin, aeruginascin, baeocystin and norbaeocystin are dephosphorylated via the same enzyme—AP. Lipophilicity of baeocystin and norbaeocystin is found to be comparable to psilocybin and their ability to cross the BBB is reliant on the dephosphorylation process [90]. These findings contradict the hypothesis proposed by Sherwood et al., which suggests that baeocystin and its metabolite exhibit poor blood–brain barrier penetrability [95]. Unlike the aeruginascin, which cannot passively cross that lipid membrane either in phosphorylated or dephosphorylated form, baeocystin and norbaeocystin are able to enter the brain wherein they might exert psychoactive effects. These findings, supported by evidence provided by different studies on aeruginascin, suggest that its direct contribution to the psychoactive effects of ‘’magic’’ mushrooms is minimal [96].

The psychoactive dose of psilocybin in humans is typically estimated at 10–20 mg when administered orally, corresponding to approximately 1–3 g of dried Psilocybe mushrooms. These values are based on controlled clinical studies assessing dose-dependent perceptual, emotional, and cognitive effects [13,97,98].

The relevant tryptamine derivatives, such as psilocybin and psilocin, are structurally similar to neurotransmitter serotonin, thus magic mushrooms are most often associated with affecting the serotonin receptors [91]. Secondary and tertiary amines, such as baeocystin, norbaeocystin and psilocybin respectively, display nanomolar affinities for most 5-HT receptor subtypes, while quaternary ammonium compounds, such as aeruginascin, have weak affinity only for 5-HT2B and SERT [91,99,100].

Behavioral changes, especially modification in unconditioned behaviors, reflect the psychoactive effects of administered substances. The psilocybin-induced head twitch response in rodents, which comes from the 5-HT2A receptor activation, is taken as evidence for its hallucinogenic properties [90,99]. Whether these features correspond with its therapeutic effects is a matter of dispute [90]. In the study conducted by Rakoczy et al., [93] no head twitch responses have been recorded during 60 min of observation after the administration of aeruginascin, baeocystin or norbaeocystin [90]. The absence of behavioral response to the aforementioned tryptamine alkaloids has been reported in the other available sources as well [95,100,101]. Even though, according to these results, tryptamines may be devoid of seeming not to have hallucinogenic properties, it does not explicitly exclude them from the group of potential therapeutic agents. The antidepressant efficacy of tryptamines was verified in this study with the Forced Swim Test (FST) in rodent models. Norbaeocystin effectiveness on immobility turned out to be akin to psilocybin, which points out its prospective antidepressant properties [90]. As mentioned before, psychedelic tryptamines are the agonists for the 5-HT2A receptors, but they can exert various effects, depending on the signaling pathway they activate. The preferential impact on one route may lead to different behavioral effects—while a Gαq-pathway is related to hallucinogenic properties, a β-arrestin mediates the antidepressant activity of the compound. This biased signaling may explain the outcome of the head twitch response test with norbaeocystin, which identifies this substance as a potential non-hallucinogenic antidepressant [90].

Psilocybin-related tryptamines and their dephosphorylated forms are, similar to psilocin, substrates for MAO—A. The authors indicate that individuals who consume psychedelic mushrooms recreationally experience noticeable differences in subjective psychological effects; these variations depend heavily on the specific taxonomic lineage of the mushroom and its precise stoichiometric profile of minor tryptamine alkaloids [95,101]. Although this was not investigated in the analyzed reports, baeocystin could potentially induce a synergistic effect with psilocin by competing for MAO, effectively increasing the concentration of psilocin in the blood [95]. One of the studies has investigated the effects of Psilocybe argentipes extract on marble-burying behavior in mice, an established animal model for obsessive–compulsive disorder [102]. Mice were administered either a mushroom extract or an equivalent dose of pure psilocybin, and their behavior was assessed. The P. argentipes extract exhibited specific anti-compulsive properties by significantly reducing marble-burying behavior without affecting locomotor activity. This effect was observed over a dose range of 0.05–2 g/kg of P. argentipes, with a statistically significant reduction at the doses of 0.1–1 g/kg, corresponding to 23.8–238 μg/kg of psilocybin. In contrast, administration of pure psilocybin at equivalent doses did not provoke any behavioral changes. Only a subtle effect was observed at doses 0.025–1.5 mg/kg, with a relevant reduction in marble-burying behavior at a dose of 1.5 mg/kg. These findings support the “entourage effect” hypothesis, which presumes that multiple compounds present in the mushroom extract work together synergistically to exert a greater or different biological effect than a single isolated compound alone. The minor tryptamine alkaloids could play a crucial role in modulating the pharmacological effects traditionally attributed solely to psilocybin [96,102].

The treatment of mental health disorders is not only limited to targeting processes occurring within the central nervous system. As the gut microbiota strongly affects the symptoms of such diseases, intentional modulation of the microbiome might be a supportive element of the therapeutic strategy [103,104]. As the 5-HT2A receptors, distributed widely throughout the gut and peripheral tissues, are the crucial part of the gut–brain axis, the novel study suggests that psilocybin and structurally similar norbaeocystin have strong potential to influence enteric processes and therefore exhibit a dual antidepressant effect [104]. Although, in the animal model trial, neither of these tryptamine alkaloids significantly affected overall microbiome variety; some measurable changes in bacterial prevalence, relevant to the dose of the tryptamine and the time since substance administration, were registered. Further studies are required to elucidate the mutual interactions between tryptamines and the gut microbiota, which could serve as an adjunct in the therapy of mental disorders.

Despite the robust emerging clinical evidence for psilocybin-assisted therapy—which in landmark head-to-head Phase II trials against escitalopram demonstrated comparable primary antidepressant efficacy and potentially superior secondary outcomes regarding sustained well-being, psychological connectedness, and social functioning—its safety profile requires strict contextualization. Psilocybin is invariably administered within highly controlled clinical settings alongside intensive, specialized psychotherapeutic support. Furthermore, current clinical trial protocols mandate the rigorous exclusion of individuals with a personal or familial history of psychotic disorders (e.g., schizophrenia) or mania, due to the severe theoretical risk of precipitating or exacerbating psychosis or manic episodes. Therefore, the safety and tolerability of psilocybin cannot be generalized to the broader psychiatric population outside of these tightly regulated therapeutic paradigms.

### 5.3. Potential Role of Bufotenin

Bufotenine (5-hydroxy-N,N-dimethyltryptamine) is a natural tryptamine alkaloid. Its structure features an indole ring-a core shared with neurotransmitters such as serotonin and DMT-distinguished by a hydroxyl group (-OH) at the 5-position and two methyl groups (-CH_3_) on the terminal nitrogen. While present in certain plants, mammals, and fungi (notably the Amanita genus), bufotenine is most famously associated with Bufo toads. The alkaloid is concentrated in their venom, eggs, and skin glands [105], making these the primary sources for pharmacological and chemical research [106].

A 2017 study found that bufotenin from the parotid gland of Bufo toads had significantly greater affinity for neuronal α7 nicotinic acetylcholine receptors than for muscle-type cholinergic receptors. Considering that the α7 receptor plays a role in long-term memory function in the brain, the study’s findings may suggest a potentially beneficial neuroprotective effect of bufotenin on the brain [107].

The analgesic effects of the alkaloid are also supported by a study published by Wang et al. [108], which demonstrated that bufotenine and several of its synthetic derivatives produced strong antinociceptive activity in a formalin-induced pain model in mice. Computational analysis indicated that bufotenin may interact with α_4_β_2_ nicotinic receptors and acetylcholinesterase (AChE), suggesting its potential in the treatment of neuropathic pain [109].

Research on the analgesic and anti-inflammatory properties of bufotenin in animal models utilizes lipidomic analysis to form new hypotheses. One such hypothesis involves its potential role in downregulating inflammatory mediators from cyclooxygenase (COX), lipoxygenase (LOX), cytochrome P450 (CYP450), linoleic acid (LA), docosahexaenoic acid (DHA), and other pathways [108]. This alkaloid may also interact with other neurotransmitters, such as dopamine and noradrenaline, which could influence its action in the nervous system. However, the mechanisms behind these interactions require further investigation.

Analysis of the psychoactive properties of bufotenin shows its ability to bind to and activate serotonin 5-HT receptors, particularly 5-HT2A and 5-HT2C. Unfortunately, despite demonstrating relatively high receptor affinity, its capacity to cross the blood–brain barrier is fundamentally limited. This restricted penetration may account for the absence of prominent psychedelic effects reported in human studies. Conversely, specific physiological studies indicate that compounds structurally analogous to bufotenine may, under specific physiological states or compromised barrier conditions, successfully traverse the blood–brain barrier [110].

The compound’s interaction with serotonin receptors induces significant changes in perception, cognition, and mood. Rick Strassman, a pioneer in modern tryptamine research, was among the first to highlight the profound impact of these molecules on human consciousness and the central nervous system [111]. Modern clinical data increasingly support this potential. In a systematic review, Vargas demonstrate that indolealkylamines may play a crucial role in treating neurological disorders [112]. Their research emphasizes the therapeutic potential of bufotenine and related compounds in managing depression, anxiety, and chronic pain, primarily through the stimulation of neuroplasticity [109].

However, in the 1960s some interesting research was published [113]. It stated that in certain psychotic disorders (e.g., schizophrenia), bufotenin may increase the risk of symptom exacerbation if not properly managed. The study found that individuals with such psychotic disorders already have bufotenin present in their body. In this context, schizophrenic individuals have displayed elevated levels of bufotenin in urine, especially during acute and untreated phases [113]. Bufotenin excretion is also observed in autistic patients with intellectual disabilities and epilepsy, as well as in most patients with depression [114]. Increased bufotenin excretion does not appear to be linked to any specific medication use by the individuals, hospitalization or dietary choices [113]. However, today’s analysis does not confirm earlier studies that suggested the presence of bufotenine in the urine during the illness. While recent findings do not support earlier reports of bufotenine in patient urine, this hypothesis remains a compelling area for research. Revisiting these studies with modern methodology could resolve existing contradictions in the data.

Although found in smaller amounts in mushrooms, bufotenin may have therapeutic potential, particularly considering its interaction with serotonin receptors. However, its effects in humans are generally weaker compared to other psychoactive substances such as psilocybin or DMT [113]. It may hold interesting therapeutic promise in treating certain neurological disorders, particularly in the context of pain relief and mood modulation. Nevertheless, due to the limited number of clinical studies and the potential risk of adverse effects, its therapeutic use requires further, detailed research.

### 5.4. Toxicological Profile and Limited Therapeutic Viability of Ibotenic Acid and Muscimol

*Amanita muscaria* (fly agaric) and Amanita pantherina contain the psychoactive isoxazole alkaloids muscimol and ibotenic acid [114,115]. While these compounds have been historically recognized for their mind-altering properties, their clinical application in modern psychiatry is highly restricted due to profound toxicological concerns and an unpredictable, narrow safety profile [115,116].

Pharmacodynamically, ibotenic acid acts as a powerful, non-selective agonist of NMDA glutamate receptors [114]. It exerts intense excitatory effects that can rapidly progress to severe excitotoxicity. In experimental neurobiology, ibotenic acid is specifically injected into rodent brains to intentionally induce localized neurochemical lesions and simulate Alzheimer’s disease-like neurodegeneration due to its potent neurotoxic properties. Muscimol, formed via the decarboxylation of ibotenic acid, acts conversely as a potent GABA-A receptor agonist, functioning as a profound central nervous system depressant [114,116].

The ingestion of these compounds induces a toxidrome recognized as pantherina-muscaria syndrome [114,116]. According to retrospective data from poison control centers, clinical manifestations typically emerge within 30 min to 2 h post-ingestion [117]. Intoxication is characterized by a dangerous and unpredictable oscillation between central nervous system excitation (manifesting as delirium, agitation, visual and auditory hallucinations, myoclonus, and occasionally seizures) and severe CNS depression (leading to lethargy, obtundation, and comatose sleep) [114,116]. Gastrointestinal distress, including significant nausea and vomiting, is also frequently reported [114]. The estimated median lethal doses (LD50) of both ibotenic acid and muscimol in murine models are significantly lower than those of most commonly prescribed psychotropic drugs, indicating an exceedingly narrow therapeutic index.

Due to the high risk of severe intoxication, the extreme variability of alkaloid concentrations across natural fungal specimens (influenced by season and geography), and a complete absence of controlled human clinical trials supporting their efficacy for mental health disorders, neither *Amanita muscaria* extracts nor isolated ibotenic acid/muscimol can be currently considered viable or safe alternatives to conventional psychiatric pharmacotherapy [115,116]. Their status remains strictly toxicological and experimental [115].

### 5.5. Ergothioneine—Properties, Mechanisms of Action, and Therapeutic Potential

Ergothioneine (ERGO) is a natural sulfur-containing amino acid derived from histidine. At physiological pH, it mainly exists in a stable thione form, which makes it highly resistant to chemical factors, high temperature, and autoxidation. Owing to these highly stable chemical properties, ergothioneine exhibits low basal reactivity while fulfilling a potent cytoprotective physiological role. The structural presence of a functional sulfhydryl group facilitates the direct neutralization of reactive oxygen species, thereby preserving cellular integrity against oxidative stress. Furthermore, ERGO demonstrates robust anti-inflammatory and detoxifying properties [17,118]. Biochemically, its synthesis involves the preliminary methylation of histidine to yield the intermediate hercynine, followed by the enzymatic conjugation of a sulfur moiety derived from cysteine to synthesize the final ergothioneine molecule [119,120].

Humans cannot produce ergothioneine on their own, so it must be obtained from their diet. The richest sources are mushrooms [17,121]. High concentrations have been found in king bolete (*Boletus edulis*), king oyster mushroom (*Pleurotus eryngii*), beech mushroom (*Hypsizygus marmoreus*), enoki (*Flammulina velutipes*), shiitake (*Lentinula edodes*), and also in brown button mushrooms (*Agaricus bisporus*) [122,123]. Ergothioneine has been recognized as safe for human consumption, including for pregnant women and children. This decision was made by the European Commission based on the opinion of the European Food Safety Authority (EFSA) [124].

Despite being highly hydrophilic, ERGO is efficiently absorbed in the digestive system and transported to various organs. This is possible due to a special transport protein called OCTN1 [121]. This transporter is found in the intestines, kidneys, liver, brain, eyes, lungs, reproductive system, and bone marrow. The highest amounts of ergothioneine are found in tissues that are most exposed to oxidative stress and inflammation [17,121]. Animal studies have shown that without the OCTN1 transporter, the body cannot store ERGO effectively in tissues [121]. Although ergothioneine is not officially considered a vitamin—because a vitamin must cause a deficiency disease when lacking—lower levels of ERGO have been linked to certain health conditions. However, it is not yet clear whether this is a cause or a result of the disease [7,121].

Human clinical trials typically use ergothioneine doses ranging from 5 to 25 mg/day. These doses have demonstrated reductions in oxidative stress markers, improvements in mitochondrial function, and potential neuroprotective effects relevant to psychiatric disorders [17,125].

Reduced levels of ERGO have been observed in individuals with mild cognitive impairment (MCI) and in patients with Parkinson’s disease (PD) and Alzheimer’s disease (AD). In people with PD, serum ERGO levels were up to 55% lower than in the control group [17,126,127]. A population study conducted in Singapore in 2016 showed that older adults with cognitive decline had significantly lower ERGO levels compared to peers with normal cognitive function. Further analysis confirmed that ERGO levels continued to drop as dementia progressed. One study also found that low plasma ERGO was associated with a 19% higher risk of MCI progressing to AD within two years [125]. ERGO shows a wide range of neuroprotective effects, including protection against oxidative stress, neurotoxicity, and neurodegeneration [17,125,128]. Since it accumulates in mitochondria, it helps protect nerve cells from damage caused by excitotoxins (like NMDA) and chemotherapeutic agents such as cisplatin and oxaliplatin.

In animal studies, oral ERGO supplementation restored memory and learning abilities impaired by chemotherapy, reduced peripheral neuropathy symptoms, and lowered acetylcholinesterase (AChE) activity [125,127]. ERGO also helps prevent the buildup of β-amyloid in the hippocampus, protects neurons from apoptosis, and reduces lipid peroxidation, supporting the brain’s redox balance. These effects were seen in animals with both normal and low levels of endogenous ERGO. In addition, ERGO protected mice from memory and learning impairments caused by D-galactose, a compound that induces accelerated brain aging.

A lack of ERGO has also been linked to reduced neurogenesis in the hippocampus, fewer dendritic spines, and poorer cognitive performance [125,129]. Regarding neuron development, ERGO supports neurogenesis, especially in areas involved in synaptic plasticity such as the hippocampus. It increases the number of dendritic spines, supports the maturation of neurons from stem cells, and activates signaling pathways like mTOR and TrkB, which may also explain its possible antidepressant effects [129,130]. Preclinical studies have shown that ERGO supplementation reduces depressive-like behavior in animals. Oral ERGO intake shortened immobility time in behavioral tests (e.g., forced swim test), and in models of social stress, it prevented depression-related behavior and sleep disturbances [130,131].

In summary, ergothioneine is a diet derived antioxidant involved with brain protection and inflammation control. Ergothioneine is primarily from mushrooms and is actively transported to organs exposed to high oxidative stress, such as the brain. Decreased ERGO levels are observed in patients with cognitive decline, Parkinson’s disease, and Alzheimer’s disease, with decreases up to 55 percent reported in Parkinson’s patients. Animal studies suggest that ERGO supplementation may be a promising neuroprotective compound, supporting neurogenesis, memory, and mood regulation. It may have potential for the prevention and management of neurodegenerative diseases and mood disorders; however, its clinical efficacy warrants further investigation. Such findings suggest that alternative supportive strategies need to be considered alongside standard treatment for brain health in the long term (Table 4).

However, more clinical studies are needed to demonstrate its efficacy, and to determine how it acts in humans, despite promising preclinical findings.

## 6. Current Limitations and Challenges in the Clinical Translation of Natural Therapies

While natural compounds present fascinating pharmacological paradigms, their integration into mainstream psychiatric practice faces substantial translational hurdles that currently preclude them from acting as immediate, standalone substitutes for conventional pharmacotherapy.

Firstly, with the notable exception of psilocybin and *Hypericum perforatum*, the vast majority of mushroom-derived and botanical compounds lack large-scale, long-term randomized controlled trials (RCTs). Much of the scientific enthusiasm surrounding agents like *Hericium erinaceus*, ergothioneine, or minor tryptamines relies on translational leaps from in vitro molecular assays and rodent behavioral models [129,130]. These preclinical models, while invaluable for establishing mechanisms of action such as neuroprotection or neurotrophic factor stimulation, frequently fail to accurately predict human central nervous system responses or complex psychiatric outcomes in real-world clinical settings [132].

Secondly, natural preparations suffer from profound standardization and quality control issues. The concentration of active secondary metabolites—such as hericenones, muscimol, or hyperforin—varies exponentially depending on geographic origin, harvest season, drying processes, and extraction methodologies [115,117]. This inherent biological variability renders precise clinical dosing exceedingly difficult and significantly increases the dual risks of under-dosing (leading to therapeutic failure) or over-dosing (precipitating toxicity).

Finally, severe regulatory and legislative hurdles persist. Hallucinogenic tryptamines are classified as Schedule I controlled substances in many international jurisdictions, drastically limiting broad clinical access, inflating the cost of research, and complicating real-world implementation outside of specialized clinics. Conversely, many non-psychoactive natural therapies (such as *Hericium erinaceus* extracts) are marketed globally as loosely regulated dietary supplements rather than stringent pharmaceuticals [117]. This pathway bypasses the rigorous, mandatory pharmacovigilance required to detect rare but severe adverse events over time, further obscuring their true long-term safety profiles.

## 7. Conclusions

The findings presented in this review demonstrate that conventional pharmacological treatments and natural bioactive compounds currently represent distinct, albeit potentially complementary, therapeutic modalities in the management of psychiatric disorders. Classical psychotropic medications remain the absolute, evidence-based standard of care, offering highly validated efficacy in preventing severe psychiatric outcomes, despite being associated with a well-documented spectrum of adverse effects that can compromise long-term treatment adherence.

In contrast, selected natural preparations, particularly psilocybin and *Hericium erinaceus*, offer promising mechanisms of action, such as rapid neuroplasticity enhancement and neurotrophic factor stimulation. However, the current evidence base for natural therapies is highly heterogeneous. While psilocybin-assisted psychotherapy is emerging as a rigorously tested, breakthrough clinical intervention for treatment-resistant depression and major depressive disorder, other compounds discussed in this manuscript—such as bufotenine, ibotenic acid, and muscimol—remain strictly investigational, preclinical, or pose severe acute toxicological risks that currently completely negate their therapeutic utility.

Furthermore, it is inaccurate to assume that all natural products are inherently safe or inert regarding drug–drug interactions. For instance, while some compounds are excreted unchanged, others, like *Hypericum perforatum*, act as potent inducers of specific Cytochrome P450 enzymes (e.g., CYP3A4, CYP2C9), precipitating dangerous metabolic interactions with established psychiatric and somatic drugs.

At present, the available clinical data do not support replacing established conventional pharmacotherapy with investigational natural preparations outside of rigorously controlled clinical trials. Future research must prioritize high-quality randomized controlled trials, strict standardization of fungal and botanical extracts, and robust toxicological profiling. The most viable path forward for modern psychiatry lies not in replacing conventional medicine with natural alternatives, but in the careful, evidence-based integration of specific, highly validated natural compounds as adjunctive therapies, tailored to individualized patient needs under strict clinical supervision.

## Figures and Tables

**Table 1:** Compound-specific comparison of natural agents evaluated for psychiatric and neurological applications.

Compound	Natural Source	Primary Mechanism of Action	Proposed Psychiatric Indication	Level of Evidence	Main Limitations & Adverse Effects	Regulatory & Safety Status
Psilocybin/Psilocin	*Psilocybe* spp.	5-HT2A receptor partial agonism; neuroplasticity induction	Treatment-resistant depression (TRD), Major Depressive Disorder (MDD)	Emerging clinical (Phase II/III RCTs)	Transient anxiety, altered perception; strictly contraindicated in psychotic disorders.	Investigational/Schedule I Controlled Substance
Hericenones/Erinacines	*Hericium erinaceus*	Stimulation of Nerve Growth Factor (NGF) and BDNF synthesis	Cognitive impairment, depressive symptomatology	Preliminary clinical/Extensive In vivo	Mild gastrointestinal distress; lack of large-scale, long-term RCTs.	Dietary supplement/Generally Unregulated
Muscimol/Ibotenic Acid	*Amanita muscaria*	GABA-A agonism (Muscimol); NMDA receptor agonism (Ibotenic acid)	None established clinically	Preclinical/Toxicological	Delirium, hallucinations, seizures, severe excitotoxicity (ibotenic acid), coma.	Highly toxic/Unregulated
Bufotenine	*Amanita* spp., *Bufo* toads	5-HT2A/5-HT2C receptor agonism	None established clinically	Preclinical/Mechanistic	Poor blood–brain barrier penetration; toxicity and autonomic side effects.	Controlled substance
Ergothioneine	Various mushrooms (e.g., *Boletus edulis*)	OCTN1-transported potent intracellular antioxidant	Cognitive decline, neuroprotection, stress-related disorders	Observational/In vivo	No established acute psychiatric efficacy; acts primarily as a nutritional protectant.	Dietary supplement (GRAS status)
Hyperforin/Hypericin	*Hypericum perforatum* (Medicinal Plant)	Non-selective reuptake inhibition (Serotonin, Dopamine, NE)	Mild-to-moderate depression	Established clinical	Severe CYP450 enzyme induction; high risk of serotonin syndrome when combined with SSRIs.	Regulated herbal medicine

Abbreviations: 5-HT2A—5-Hydroxytryptamine 2A receptor; 5-HT2C—5-Hydroxytryptamine 2C receptor; BDNF—Brain-Derived Neurotrophic Factor; CYP450—Cytochrome P450 enzyme system; GABA-A—Gamma-aminobutyric acid type A receptor; GRAS—Generally Recognized As Safe; MDD—Major Depressive Disorder; NE—Norepinephrine; NGF—Nerve Growth Factor; NMDA—N-methyl-D-aspartate receptor; OCTN1—Organic Cation/Carnitine Transporter 1; RCTs—Randomized Controlled Trials; SSRIs—Selective Serotonin Reuptake Inhibitors; TRD—Treatment-Resistant Depression.

**Table 2:** Summary of cohort studies examining antidepressant effectiveness and adverse effects.

Examined Group	Medication and % of Patients Who Used It	Adverse Effects and Other Problems	Reference
673,177 patients with depression	SSRIs (85.7%)—fluoxetine, citalopram, escitalopram, fluvoxamine, paroxetine, sertraline, TCAs (6.9%)—amitriptyline, clomipramine, dosulepine, doxepine, imipramine, lofepramine, maprotiline, mianserin, nortriptyline, trimipramine, MAOIs (0.01%)—moclobemide, phenelzine, others—agomelatine, duloxetine, mirtazapine, nefazodone, reboxetine, trazodone, venlafaxine, vortioxetine	- 45.6% suffered at least one adverse effect - at 2 months 38.6% of people discontinued the treatment and 5.6% of people did that due to an adverse effect - at 12 months 87.7% of people discontinued the treatment and 12.5% of people did that due to an adverse effect The most common side effects included: nausea, vomiting, diarrhea, constipation, headache, dizziness, insomnia, sexual dysfunction, fatigue, anxiety, heart rhythm disturbances, increased blood pressure, suicidal thoughts, self-harm, emotional lability	[9]
238,963 patients aged 20–64	SSRIs (79.5%), TCAs (25.9%)	- during 5-year follow-up: falls (1.96%), fractures (2%), upper gastrointestinal bleeding (0.44%), traffic accident (1.54%), adverse drug reaction (0.44%)	[33]
222,121 aged 40–69	SSRIs	- hazard ratio consecutively at 5 and 10 years: cerebrovascular disease (1.12 and 1.34), congenital heart disease (1.44 and 1.15), cardiovascular disease mortality (1.16 and 1.87)	[34]
100 adults with depression (49%), panic disorder (14%), OCD (13%)	SSRIs	- flatulence (64%), somnolence (59%), memory impairment (51%), decreased concentration (50%), yawning (47%), fatigue (45%), dry mouth (45%), weight gain (45%), light headedness (43%), sweating (38%)	[35]
1431 adults aged 18–78	SSRIs (75.7%), TCAs (10.9%)	- emotional numbness (66.1%), feeling foggy/detached (65.1%), sexual difficulties (64.2%), drowsiness (59.6%), weight gain (55.8%), insomnia (55.7%), suicidality (42.5%), feeling aggressive (37.6%), feeling of addiction (36.8%) and others	[10]

Abbreviations: MAOIs—monoamine oxidase inhibitors; SSRIs—selective serotonin reuptake inhibitors; TCAs—tricyclic antidepressants.

**Table 3:** Most commonly used medicinal plants in psychiatry.

Medicinal Plant	Main Active Compounds	Mechanisms of Action	Potential Therapeutic Effects	Reference
*Hypericum perforatum* (St John’s wort)	Hypericin, hyperforin, flavonoids	Inhibition of serotonin, norepinephrine, and dopamine reuptake; anti-inflammatory and antioxidant effects	Mild to moderate depressive episodes; mood improvement	[62,64]
*Valeriana officinalis* (valerian)	Valerenic acid, valepotriates	Modulation of ${GABA}_{A}$ receptors; sedative effects	Anxiety, insomnia, nervous tension	[65]
Passiflora incarnata (passionflower)	Flavonoids (vitexin), harmala alkaloids	GABA modulation; anxiolytic effects	Generalized anxiety, tension, sleep disturbances	[66]
Lavandula angustifolia (lavender)	Linalool, linalyl acetate	Modulation of GABA and glutamate; calming and anxiolytic effects	Anxiety disorders, stress, tension	[67]
Rhodiola rosea (rhodiola)	Rosavins, salidroside	Regulation of the HPA axis; adaptogenic effects; increased serotonin and dopamine	Stress, fatigue, mild depressive symptoms	[68]
Withania somnifera (ashwagandha)	Withanolides	GABA modulation, cortisol reduction, anti-inflammatory effects	Anxiety, stress, adjustment disorders	[69]

Abbreviations: GABA—gamma-aminobutyric acid; ${GABA}_{A}$—gamma-aminobutyric acid type A receptor; HPA axis—hypothalamic–pituitary–adrenal axis.

**Table 4:** Evidence summary distinguishing robust clinical trials from preclinical/observational data.

Compound	Study Design & Population	Intervention/Dose	Comparator	Main Findings	Safety Findings & Limitations	References
Psilocybin	Phase II Human RCT (MDD, *n* = 59)	Psilocybin (25 mg × 2 sessions, 3 weeks apart)	Escitalopram (10–20 mg/day for 6 weeks)	Comparable primary antidepressant effect (QIDS-SR-16); psilocybin superior in secondary well-being and functioning measures	Favorable tolerability profile. Requires intensive therapy support; patients with severe somatic disease or psychosis excluded	[114]
*Hericium erinaceus*	Double-blind Human RCT (MCI, *n* = 30) and young adult cohorts	Standardized extract (up to 3000 mg/day)	Placebo	Significant improvement in specific cognitive functions (e.g., Stroop task) and potential mood enhancement	Small sample sizes; long-term efficacy, sustained effects post-discontinuation, and optimal dose–response remain unestablished	[117]
Psilocybin-related minor tryptamines	Animal Model (In vivo, rodents)	Norbaeocystin, aeruginascin	Saline/Psilocybin	Norbaeocystin reduced immobility in Forced Swim Test without inducing a 5-HT2A-mediated head-twitch response	Purely preclinical data; human pharmacokinetics, efficacy, and safety remain completely unverified	
Muscimol/Ibotenic Acid	Human Observational (Poison Control Registry)	Unregulated oral ingestion of *Amanita muscaria*	N/A	Rapid onset of CNS excitation followed by profound CNS depression and GI distress; potential for coma and seizures	Severe acute toxicity; potential lethal outcomes; no psychiatric therapeutic index clinically established	[115,116]
Ergothioneine	Animal Model (In vivo, mice/transgenic APP/PS1)	Oral ERGO supplementation	Vehicle/Stress models	Synergistic neuroprotective effects and amelioration of cognitive deficits and depressive-like behavior following stress	Purely preclinical behavioral data; direct translation to human psychiatric conditions is pending robust RCTs	[132]

## Data Availability

No new data were created or analyzed in this study. Data sharing is not applicable to this article.

## Abbreviations

The following abbreviations are used in this manuscript:

DSM 5	Diagnostic and Statistical Manual of Mental Disorders, Fifth Edition
ICD 11	International Classification of Diseases 11th Revision
OCD	obsessive–compulsive disorder
PTSD	Post-traumatic stress disorder
SSRIs	Selective Serotonin Reuptake Inhibitors
SNRIs	Serotonin-Norepinephrine Reuptake Inhibitors
TCA	Tricyclic Antidepressants
5 HT2A	5 Hydroxytryptamine 2A receptor
BDNF	Brain Derived Neurotrophic Factor
CYP450	Cytochrome P450 enzyme system
GI	Gastrointestinal
MAOIs	Monoamine Oxidase Inhibitors
NGF	Nerve Growth Factor
NMDA	N Methyl D Aspartate receptor
GABA	Gamma-Aminobutyric Acid
GABAA	Gamma-Aminobutyric Acid type A receptor
RCT	Randomized Controlled Trial
HPA axis	Hypothalamic–Pituitary–Adrenal axis
CNS	Central Nervous System
PD	Parkinson’s Disease
TCIM	Traditional, complementary, and integrative medicine
DDD	defined daily doses
NASSAs	noradrenaline and specific serotonergic antidepressants
LTLT	long-term lithium therapy
ADHD	attention deficit hyperactivity disorder
ALS	amyotrophic lateral sclerosis
MS	multiple sclerosis
LPS	lipopolysaccharide
CES-D	Center for Epidemiologic Studies Depression Scale
AP	alkaline phosphatase
BBB	blood–brain barrier
FST	Forced Swim Test
COX	cyclooxygenase
LOX	lipoxygenase
LA	linoleic acid
DHA	docosahexaenoic acid
ERGO	Ergothioneine
MCI	mild cognitive impairment
PD	Parkinson’s disease
AD	Alzheimer’s disease
AChE	Acetylcholinesterase
